# Comparison of the Effects of Essential Oils from *Cannabis sativa* and *Cannabis indica* on Selected Bacteria, Rumen Fermentation, and Methane Production—In Vitro Study

**DOI:** 10.3390/ijms25115861

**Published:** 2024-05-28

**Authors:** Aleksandra Tabiś, Antoni Szumny, Jacek Bania, Katarzyna Pacyga, Kamila Lewandowska, Robert Kupczyński

**Affiliations:** 1 Department of Food Hygiene and Consumer Health Protection, Wrocław University of Environmental and Life Sciences, 50-375 Wrocław, Poland; aleksandra.tabis@upwr.edu.pl (A.T.); jacek.bania@upwr.edu.pl (J.B.); 2 Department of Food Chemistry and Biocatalysis, Faculty of Biotechnology and Food Sciences, Wrocław University of Environmental and Life Sciences, 50-375 Wrocław, Poland; antoni.szumny@upwr.edu.pl; 3 Department of Environment Hygiene and Animal Welfare, Faculty of Biology and Animal Science, Wrocław University of Environmental and Life Sciences, 50-375 Wrocław, Poland; katarzyna.pacyga@upwr.edu.pl (K.P.); kamila.lewandowska@upwr.edu.pl (K.L.)

**Keywords:** hemp, cows, volatile fatty acids, essential oils, rumen fermentation, methane

## Abstract

This study aimed to evaluate the effects of essential oils (EOs) extracted from *Cannabis sativa* L. and *Cannabis indica* Lam. on in vitro ruminal fermentation characteristics, selected rumen microbial populations, and methane production. GC-MS analyses allowed us to identify 89 compounds in both EOs. It was found that *E*-β-caryophyllene predominated in *C. sativa* (18.4%) and *C. indica* (24.1%). An in vitro (Ankom) test was performed to analyse the control and monensin groups, as well as the 50 µL or 100 µL EOs. The samples for volatile fatty acids (VFAs), lactate, and microbiological analysis were taken before incubation and after 6 and 24 h. The application of EOs of *C. indica* resulted in an increase in the total VFAs of acetate and propionate after 6 h of incubation. The applied EOs had a greater impact on the reduction in methane production after 6 h, but no apparent effect was noted after 24 h. Lower concentrations of *C. sativa* and *C. indica* had a more pronounced effect on *Lactobacillus* spp. and *Buryrivibrio* spp. than monensin. The presented findings suggest that *C. sativa* and *C. indica* supplementation can modify ruminal fermentation, the concentrations of specific volatile fatty acids, and methane production.

## 1. Introduction

*Cannabis sativa* L. is a plant that has been known since ancient times and is used for various purposes, including medical and even religious. At present, it continues to constitute one of the most important groups of raw materials used for the preparation of infusions and additives in the food and beverage industry, as well as in the nutraceutical and pharmaceutical sectors, due to its varied chemical composition [1,2]. This herb represents a rich source of secondary metabolites, including terpenoids, flavonoids, alkaloids, lignans, and fatty acids, among others [3]. Based on its content, *Cannabis sativa* L. is classified into two types, namely, medical and industrial. The first group, also known as marijuana, includes plants with very high levels of δ-9-tetrahydrocannabinol (THC) (0.3–38% of the dry weight), while the second group, called hemp, comprises plants with a very low THC content (0–0.3% of the dry weight) [4,5,6]. Genetic and plant breeding studies have allowed for the acquisition of varieties characterised by an absence of THC or very low THC content (<0.2%), which are generally known as fibre hemp, industrial hemp, or oil hemp [7,8].

In recent years, there has been a growing interest in the essential oils (EOs) extracted from low-THC types of *Cannabis sativa* L. [9,10]. The most abundant EOs, secreted by the glandular trichomes found in inflorescences and leaves, include α-pinene, β-myrcene, and α-terpinolene (among other monoterpenes), together with β-caryophyllene and α-humulene (among other sesquiterpenes) [9]. The profile of the essential oils depends on the genotype of the plant and the growth conditions, as well as the extraction method. To isolate these bioactive compounds, several approaches (e.g., using steam, water, carbon dioxide, or organic solvents) can be employed. One of the simplest and most widely applied techniques is hydrodistillation [11], which is considered environmentally friendly and, in addition, requires low investment and operating costs [12]. Furthermore, it can be incorporated into the manufacture of numerous certified bioproducts [11]. Increased attention to hemp essential oils is associated with their wide spectrum of biological activities, such as antibacterial, antifungal, antiviral, antiparasitic, repellent, antioxidant, anti-inflammatory, anticancer, insecticidal, and allelopathic activities [1,7,13,14,15,16,17,18,19]; thus, they can be applied in a vast number of industries around the world.

Therefore, essential oils can be used in almost all fields of life as natural alternatives to conventionally available products or therapies. EOs have been used for centuries in ethno-veterinary practices and animal health management. The most widespread practices include: the application of pine-derived EOs to treat ectoparasites and to disinfect wounds; camomile and yarrow to cure inflammations; and anise, caraway, and fennel to prevent gastrointestinal problems (i.e., colic and flatulence) [20]. However, the evolution of Western medicine has led to the replacement of plants, extracts, and volatile oils with synthetic chemicals in mainstream animal healthcare [20,21]. On the one hand, the application of antibiotics, hormones, and man-made pharmaceuticals provides many benefits, including health maintenance, as well as higher-quality livestock with greater productivity [22]. On the other hand, the widespread usage of such synthetic chemicals, especially in intensive animal production, has caused numerous adverse effects, of which one of the most perilous is the spread of antibiotic resistance and, thus, a reduction in the availability of compounds effective for animal pathogenic bacteria [21]. The growing public awareness of threats to animal and human health, along with environmental safety, has contributed to a significant intensification of efforts to reduce the risks associated with antibiotic use [20]. For this reason, a return to natural treatments that use herbs and herbal products is proposed as one of the most promising alternatives to xenobiotics [20]. Furthermore, hemp-based ingredient supplementation in livestock has been found to reduce stress and inflammation, further affecting their behaviour (e.g., by improving lying behaviour) [23].

Hemp essential oils can be administered in liquid or encapsulated form. Studies carried out on human keratinocytes, fibroblasts, and bronchial epithelial cells showed differences in cell toxicity and cytokine expression, depending on their method of application [24]. Of particular relevance is the possibility of implementing EOs in the dairy industry as a promising alternative to ionophores, due to their potential for limiting antibiotic consumption [25]. In most cases, when the concentration of the active ingredient in in vitro fermentation tests of rumen fluid exceeds 500 mg·L^−1^, adverse effects are observed. At moderate doses (from 50 to 500 mg·L^−1^, depending on the active compound), certain essential oils and their active ingredients are capable of modifying rumen fermentation by altering volatile fatty acid production, protein metabolism, or both [13]. The study by Winders et al. [26] showed that the addition of hempseed cake, which makes up 20% of the diet of beef heifers, resulted in enhanced microbial diversity in the rumen, decreased microbial richness in the vagina, and increased diversity and richness in the uterus. Alterations in the bovine gut, respiratory system, and reproductive microbiota were observed. One of the most recent studies has indicated that EO dietary supplementation does not affect lactation performance or methane emission rates, but can reduce the intensity of methane production per kg of milk fat by 8.5% [27]. Other reports show that methane emissions can be reduced by 12% after EO application but note that the impact can be temporary, with weekly changes [28].

This is the first study on the effects of EOs obtained from various varieties of *Cannabis* on the population of rumen bacteria responsible for the synthesis of conjugated linoleic acid (CLA) isomers and the bacteria dominant in rumen acidosis (*Streptoccocus bovis* and *Lactobacillus* spp.). This study aimed to evaluate the effects of essential oils derived from *Cannabis sativa* L. and *Cannabis indica* Lam. on in vitro ruminal fermentation characteristics and total gas and methane production. The hypothesis posed in our research is that EOs extracted from various species of *Cannabis* can reduce the predominant bacteria characteristics of rumen acidosis and can have a targeted impact on total VFAs, as well as individual compounds.

## 2. Results

### 2.1. Analysis of Essential Oils

This is the first study investigating the effects of essential oils obtained from *C. sativa* and *C. indica*, sources that are rich in mono- and sesquiterpenoids, on in vitro rumen fermentation. GC-MS analyses allowed for the identification of 89 compounds (Table 1). The quantitative and qualitative compositions of the essential oils were typical of these species. It was found that *E*-β-caryophyllene and β-myrcene were two compounds that predominated in *C. sativa*. In addition, limonene, α-pinene, α-humulene, and caryophyllene epoxide occurred in amounts greater than 7%. In the case of *C. indica*, E-β-caryophyllene, along with terpinolene, predominated in the mixture. β-Ocimene, α-pinene humulene, and β-pinene occurred in the EOs in amounts greater than 5%. Ibrahim et al. [29] have reported that similar terpenoids were the main components of *C. sativa* cultivated in Washington State. Naz et al. [30] proved that the sesquiterpenoid β-caryophyllene and its oxide, as well as linalool and bergamotene, were the main constituents.

**Figure 1:**
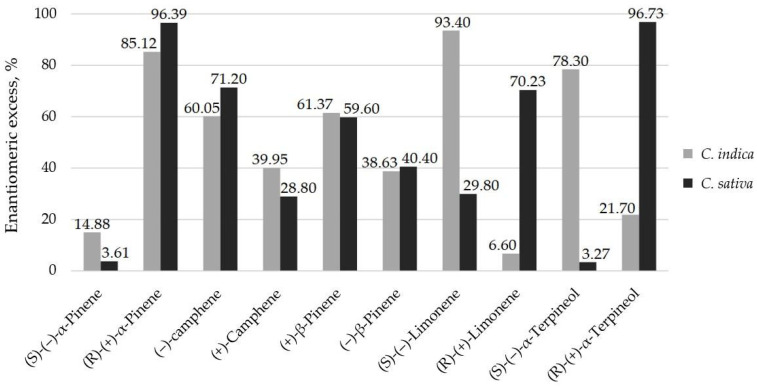
The enantiomeric excess of selected terpenes that are present in EOs.

The occurrence of differences in the enantiomeric composition of optically active compounds can affect the biological properties of essential oils. Information on the variation of the enantiomeric composition of essential oil components confirms the variation of the chemical structure of the mixture. Consequently, the enantiomeric excess of five chiral monoterpenes that occurred in the EOs was also assessed. It was found that *C. indica* was characterised by a great excess (more than 96%) of R(+) enantiomers of α-pinene and R (+)-α-terpineol. In the case of β-pinenes, no differences were observed. The enantiomeric excess of (+) enantiomers was nearly 60% (Figure 1).

**Figure 2:**
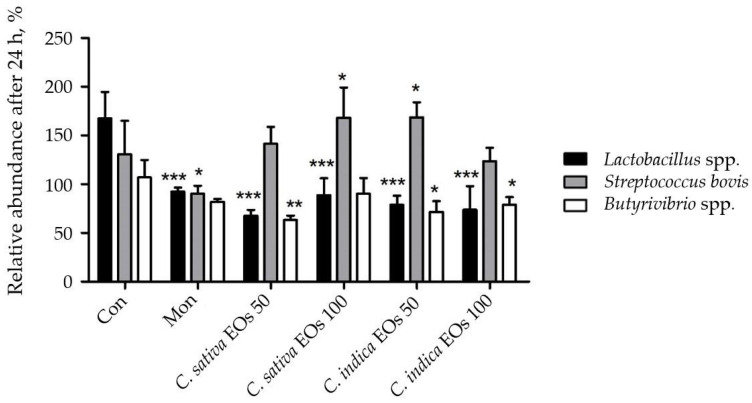
Changes in population of selected microorganisms after 24 h of incubation; data are calculated at the initial value in %. Treatments: Con—control; Mon—1 mg of monensin; *C. sativa* 50—50 µL of EO from *C. sativa; C. sativa* 100—100 µL of EO from *C. sativa*; *C. indica* 50—50 µL of EO from C. *indica*; *C. indica* 100—100 µL of EO from C. *indica*; * *p*  <  0.05, ** *p*  <  0.01, *** *p * <  0.005 vs. control group.

After 24 h of incubation of *C. sativa* and *C. indica*, a significant increase in the concentration of propionic acid was observed. A similar impact of the EOs was noted for the concentration of butyric acid, where the effect was dose-dependent. The application of *C. indica* essential oil resulted in an increase in the amount of propionic acid, in contrast to the EO from *C. sativa*. Additionally, *C. sativa* caused the greatest reduction in methane production. Moreover, enhancement of the growth of the lactic acid-producing bacteria population (*S. bovis*) was observed after the use of essential oils obtained from *C. sativa* and *C. indica* (Figure 2).

### 2.2. In Vitro Rumen Fermentation Characteristics

The impact of two volumes (50 and 100 µL) of the essential oils of the two *Cannabis* species (marked as *C. sativa* 50 and *C. sativa* 100 and *C. indica* 50 and *C. indica* 100, respectively) on the characteristics of in vitro fermentation is shown in Table 2.

The addition of EOs did not significantly affect the pH at the two examined times.

After 6 h of incubation, the treatment with *C. indica* 100 caused the highest growth in the total volatile fatty acids (VFAs), acetate, and propionate concentrations: 11.5, 15.4, and 3.0% compared to the control (Con), respectively. Regarding the monensin-containing group (Mon), increases of 11.3 and 18.3% were observed for the VFAs and acetate, respectively, but there was a slight decrease (3.6%) in propionate. In general, after a shorter incubation time, the lowest VFA content was noted in the groups with *C. indica* 50 (6.2 and 6.3% lower than in Con and Mon) and *C. sativa* 100 (5.8% and 6.0% lower than in Con and Mon). The addition of *C. indica* 50 also decreased the amount of propionate (by 31.5 and 35.9% compared to Con and Mon), while *C. sativa* 100 reduced the amount of acetate, but the reduction was not statistically significant (1.1% lower than in Con and 1.5% higher than in Mon). The butyrate content decreased in all the experimental groups. The lowest amount was in the groups with the addition of *C. indica* 50 (25.1% and 10.7% lower than in Con and Mon) and *C. sativa* 50 (23.7% and 9.0% lower than in Con and Mon).

An opposite correlation for the group treated with *C. indica* 100 was noted after 24 h of incubation—decreases in VFAs (by 19.5 and 21.7%), acetate (by 19.2% and 18.0%), and propionate (by 0.3 and 12.9%) were observed compared to Con and Mon, respectively. The greatest propionate-reducing activity was demonstrated by *C. sativa* 50 (3.8% and 16.0% lower compared to Con and Mon, respectively), while the greatest stimulation of activity was in the *C. indica* 50 group (2.4% higher than in Con and 11.8% lower than in Mon). The highest total VFA content was observed in the groups treated with *C. sativa* 100 (12.7 and 9.6% higher than in Con and Mon, respectively) and *C. sativa* 50 (11.8 and 8.7% higher than in Con and Mon, respectively). The use of *C. sativa* 50 also increased the amount of acetate by 16.1 and 17.7% compared to Con and Mon. The essential oils did not significantly affect the concentration of butyrate compared to the control group, with the exception of *C. indica* 50, which reduced its amount by 6.0%. However, an effect was observed in comparison with the monensin-treated group. For example, the EOs increased the content of butyrate by 22.0% after the use of *C. indica* 100.

The acetate and propionate ratios were higher in all the experimental groups, excluding the *C. indica* 100 group after 24 h of incubation, in which the ratio was 20.0% lower than in the control group. The treatment with *C. indica* 50 caused the greatest increase in the tested parameter after 6 h (by 49.2 and 66.2% compared to Con and Mon, respectively), followed by the treatment with *C. sativa* 50 after 24 h (by 19.2 and 38.4% compared to Con and Mon).

No statistically significant effect on the fermentation efficiency was observed.

A similar trend to that of the acetate and propionate ratio was observed for the VFA utilisation index (NGR)—an increase was noted in all the experimental groups at 6 and 24 h, with the exception of *C. indica* 200 after 24 h of incubation (12.4 and 4.5% lower than in Con and Mon, respectively). Treatment with *C. sativa* 50 resulted in increments of 24.6 and 15.1% in comparison to Con and Mon after 6 h of incubation, and of 13.3 and 23.6% after 24 h.

The methane production was higher after 24 h of incubation than after 6 h. In most of the groups, a decrease in concentration was observed after a shorter time compared to the control group, while an increase was noted compared to the monensin group. The application of *C. sativa* 100 increased methane production (by 21.9 and 58.3% compared to Con and Mon), while the use of *C. indica* 100 decreased its production (by 27.7 and 6.1% compared to Con and Mon) to the greatest extent. After 24 h, the highest level of production was observed in the groups treated with *C. sativa* 50 (44.4 and 97.3% higher than in Con and Mon) and in the *C. indica* 50 group, it was the lowest (15.2 and 57.4% higher than in Con and Mon).

The lactate concentration was lower in both the monensin-treated and essential oil-treated groups. For instance, the *C. sativa* 50 group was characterised by the lowest lactate content (11.8% lower than in Con and 8.7% higher than in Mon), while the *C. sativa* 100 group exhibited the highest concentrations (9.6% lower than in Con and 11.5% higher than in Mon).

### 2.3. DNA Extraction and Analysis of Changes in the Composition of Microorganisms Using qPCR

The results of the quantification of the target bacterial DNA are shown in Figure 2. Compared to the control, the addition of any essential oil, regardless of the *Cannabis* species, resulted in a significant decrease in the relative abundance of *Lactobacillus* spp. bacteria (*p* < 0.01). The relative abundance of *Streptococcus bovis* increased significantly when 50 µL of *C. indica* essential oil (*p* = 0.05) and 100 µL of *C. sativa* essential oil (*p* = 0.05) were added to the rumen fluid. The relative abundance of *Butyrivibrio* spp. decreased significantly after the addition of 50 µL of *C. indica* (*p* = 0.05) and *C. sativa* (*p* = 0.01) essential oil to the ruminal fluid.

The incorporation of large amounts of hemp seed meal (33% on a dry matter (DM) basis) into the diet of small ruminants (growing goats) led to a decrease in VFA concentration in the rumen, which originated from a reduction in energy supply [31]. Similarly, other researchers did not find any effect of the use of hemp seed meal on dairy cow feeding [32]. However, the increase in crude protein and gross energy supply with the addition of hemp meal raised the concentrations of ammonia in rumen and the VFAs of rumen digesta [33].

## 3. Discussion

The scientific literature shows growing interest in the use of the by-products of hemp seed processing and oil extraction as dietary ingredients in the feeding of animals [32]. However, there is less research on *Cannabis* varieties as a viable feed for ruminants than there is for non-ruminant species. Therefore, information on the effects of this potential form of nourishment on carcass characteristics, fat deposition, growth performance, and milk production is very limited [34]. As part of this discussion, due to the lack of reports on the impact of hemp essential oils on rumen fermentation and methane production, we focused on the impact of hemp by-product supplementation on ruminants. In addition, we provided some examples of the benefits that can arise from the inclusion of essential oils from various plants as natural feed additives.

The antimicrobial activity of essential oils is very well-recognised in the literature. As essential oils are most often a mixture of dozens of compounds, singling out one component as the key to their bactericidal or bacteriostatic activity is usually impossible. It is thought that the effect of EOs may be a synergy of the interaction of individual components. However, we can assume that the components making the highest contribution to biological activity may be those in oils with the highest levels of thymol and carvacrol, as well as eugenol, as these are used in practice to modulate rumen microflora activity [35]. The modulation of methane and ammonia production via rumen bacteria was found with a treatment using essential oils with a high content of caryophyllene, as has been extensively described by Cobellis et al. [36].

According to the literature, the predominant compounds in both EOs, E-β-caryophyllene, terpinolene, and myrcene, have strong antimicrobial and bacteriostatic activities. Moo et al. found that β-caryophyllene causes the intracellular leakage of *B. cereus* cells treated with this sesquiterpene at a concentration of 1.24% [37]. Moreover, an increase in the bacterial membrane permeability surface was measured. The growth and formation of *Streptococcus* strains were inhibited at a caryophyllene concentration of 0.078% [38]. 

The occurrence of differences in the enantiomeric composition of optically active compounds can affect the biological properties of essential oils. Information on variations in the enantiomeric composition of essential oil components confirms the variation in the chemical structure of the mixture. Consequently, the enantiomeric excess (ee) of five chiral monoterpenes occurred in the EOs. It was found that *C. indica* was characterised by a high level of excess (above 96%) of R(+) enantiomers of α-pinene and R (+)-α-terpineol. In the case of β-pinenes, no differences were observed. The ee of the (+) enantiomer was nearly 60%. The enantiomeric compositions of monoterpenes and monoterpenoids in essential *C. sativa* EO have been described by other authors; for example, Cucinotta et al. [39] showed that (+) isomers of pinenes predominated in a Futura 75 variety growing in Italy.

In the study presented by Araiza-Rosales et al. [40], the residues obtained after cold pressing and extraction of the flower cakes of *C. sativa* were used as a potential forage source. An in vitro assay, conducted with the use of steer inoculum, showed that the addition of plant samples produced a better utilisation of nutrients by the microorganisms present in ruminal fermentation and consequently improved the digestibility of dry matter and the fermentation parameters in vitro. However, total bacteria and N-ammonia levels were reduced, while the total volatile fatty acids increased. Furthermore, a growth in methane and CO_2_ production was observed, indicating an improvement in ruminal fermentation. In the study conducted by Hessle et al. [41], cold-pressed hempseed cake was used as a protein feed for young calves and finishing steers. The authors stated that feeding with this biomass resulted in similar weight gain and carcass traits compared to soybean meal, but improved rumen function due to a higher fibre content and/or lower starch content. Vastolo et al. [42] conducted research on the ruminal inoculum of buffaloes, and showed that hemp co-products possess valuable nutritional characteristics (the crude protein content is higher than 20% on the basis of DM, and the high neutral detergent fibre concentration is partially lignified). The parameters of in vitro gas production revealed low fermentation (as evidenced by the degradability of organic matter and the cumulative volume of gas) and methane production. The authors suggested that the CH_4_ formation process could be affected by the high amounts of lipids and ash or by the content of cannabidiolic acid (CBDA). Moreover, they hypothesise that hemp co-products could be beneficial for reducing methane production in the rumen [42]. Additionally, Jensen et al. [43] evaluated the extracts obtained from *C. sativa* for their potential to mitigate methane in an in vitro model of cow rumen fermentation. The authors showed that the extract solutions, due to the presence of flavonoid glycosides, significantly decreased methane production, with no adverse impact on feed degradability and volatile fatty acid patterns. Similar results in terms of the suppression of methane emissions after the dietary supplementation of cows with *C. sativa* oilseeds were confirmed in another study carried out by Wang et al. [44]. Interesting results on the impact of an industrial hemp ethanol extraction by-product on digestibility, lactation performance, plasma metabolites, ruminal fermentation, and bacterial communities in dairy cows were also demonstrated in one of the most recent studies [45]. As a result, dietary enrichment did not affect lactation performance but decreased the levels of total volatile fatty acids and butyrate compared to the animals in the control group. The reduction in the plasma IL-1β content was noted, and no effects on other blood parameters were observed. Furthermore, the relative abundances of *Bacteroidota*, *Fibrobacterota*, and *Prevotellaceae* in the rumen fluid increased, while *Firmicutes* tended to decline with the increasing dose. Linear growth in the relative abundances of *Firmicutes*, *Lachnospiraceae*, *Monoglobaceae*, and *Butyricicoccaceae* in faeces was noted. A favourable finding of this study was a reduction in dairy farming costs with the increasing dosage of the supplement [45]. A pilot study presented by Wróbel et al. [46] was carried out to evaluate the effect of inoculation with lactic acid bacteria starter culture on fermentation and the chemical composition of the shoots (HS) and flowers (HF) of hemp silage. In the case of the HS, the application of Lactosil Biogas to hemp caused a reduction in pH and fungal abundances but an increase in lactic acid. In the HF group, the bacterial inoculation was less effective; however, a rise in lactic acid and a decrease in butyric acid was noted. Furthermore, the ensilage process led to a decrease in crude fibre and hemicellulose in both silages [46]. The addition of hemp seed oil to the microbial communities in the rumen of dairy buffalo was researched by Zhou et al. [47]. The authors showed that the employed treatments exerted a weak effect on the total number of bacterial and fungal taxa but showed a significant increase in lactic acid. A few reports on the absence of the significant effects of *Cannabis* on animals are also available. For example, research carried out by Chatterjee et al. [48] reported no effect of *C. indica* supplementation on the total gas production, volatile fatty acid pattern, substrate degradability, and enzyme activities in an in vitro assay using rumen fluid from adult buffaloes. Similarly, other authors stated that hempseed cake is a successful substitute for soybean meal in the diet of cull dairy cows without influencing in vivo performance, carcass characteristics, and meat quality. They did not observe any impact on the FA composition of intramuscular fat (excluding DFA, which was lower) [49].

Interest in the use of essential oils as antimicrobial agents and feed supplements in animal nutrition has increased in recent years, due to the emphasis on the use of natural compounds as an alternative to feed antibiotics in animal production [13,50,51,52,53,54,55,56,57,58,59,60]. The literature provides many in vitro and in vivo reports in this field. For instance, a comprehensive study on the effects of essential oils (EOs) from various plants and their components (EOCs) on rumen microbial fermentation in vitro was carried out by Benchaar et al. [61]. The authors examined the influence of carvacrol (400 mg·L^–1^), cinnamaldehyde (400 mg·L^–1^), cinnamon leaf EO (400 mg·L^–1^), clove leaf EO (mg·L^–1^, eugenol (800 mg·L^–1^), oregano EO (200 mg·L^–1^), sweet orange EO (200 mg·L^–1^), thyme EO (200 mg·L^–1^), and thymol EO (400 mg·L^–1^). The treatments were evaluated using an in vitro batch culture of rumen fluid collected from dairy cows. Ruminal fermentation was only affected by the phenolic compounds, carvacrol, thymol, and eugenol. Carvacrol and eugenol caused an increase in pH and in the molar proportion of butyrate but a decrease in the molar proportion of propionate, dry matter in vitro, neutral detergent fibre, and gas production. The addition of thymol resulted in an increase in the final pH and a reduced molar proportion of propionate, neutral detergent fibre, and gas production. No effect of EO or EOC on NH_3_ concentration was noted. Moreover, only the phenolics displayed antimicrobial activity, which was manifested in the form of the reduced fermentability of the diet and a change in the VFA profile from less propionate to more butyrate. However, these changes may not be nutritionally favourable for livestock. The results suggest that the application of essential oils must be carefully defined before their widespread use [61]. In a study by Garcia et al. [62], the possibility of implementing essential oils extracted from *Lippia turbinata* and *Tagetes minuta* was explored to minimise methane production during the in vitro fermentation of substrates. It was found that EOs at a dose of 30 µL·L^–1^ reduced methane levels, along with an insignificant inhibition of substrate digestibility [62]. In another study, conducted by Gunal et al. [63], the effects of the addition of EOs (125, 250, 500 mg·L^–1^) obtained from anise (ANO), cinnamon (CNO), cedar wood (CWO), eucalyptus, and tea tree (TEO) on in vitro cow rumen fermentation and biohydrogenation were investigated. The authors found that EOs (with the exception of CNO) did not affect the VFA profile, the proportions of acetate and propionate, or the acetate-to-propionate ratios. Moreover, the addition of CNO, CWO, and TEO decreased the total VFA concentrations, regardless of dose level. The content of ammonia-N and linoleic acid was higher in the cultures incubated with EOs, while the concentrations of C18:0 and trans C18:1 diminished. A significant reduction in the formation of biohydrogenation products was observed [63]. An in vitro screening of the effects of EO active compounds (bornyl acetate, carvacrol, 1,4-cineole, citral, p-cymene, eugenol, limonene, linalool, α-pinene, and β-pinene) at a dose of 1 mg·L^–1^ on the fermentation characteristics of cow rumen and the production of methane was investigated. The authors stated that most of the bioactive compounds reduced methane production (by as much as 86%). It was observed that only 1,4-cineole, bornyl acetate, limonene, and α-pinene did not inhibit volatile fatty acid (VFA) production, and only bornyl acetate influenced the reduction in methane production per mol of VFA [64]. However, certain EOs could be toxic to the ruminal microbiota, due to their antimicrobial and anti-nutritional effects at larger doses [13,54]. For example, an in vitro study of limonene and thymol at higher concentrations exhibited activity that was toxic to rumen bacteria [54].

*Streptococcus bovis* and *Lactobacillus* spp. are starch- and sugar-fermenting species that mainly produce lactate; therefore, they are considered to be associated with SARA and acute ruminal acidosis [65]. The studies carried out by our team showed a significant decrease in the relative abundance of *Lactococcus* spp. compared to the control in the case of both *C. sativa* and *C. indica*, regardless of the amount added. The relative abundance of *S. bovis* did not change (*C. indica* 100 and *C. sativa* 50) or increase significantly (*C. indica* 50 and *C. sativa* 100), compared to the control 24 h after the start of incubation. *Butyrivibrio* species can degrade hemicelluloses and they also have the ability to biohydrogenate fatty acids [66]. In contrast to the results in [47], no significant positive correlation was observed between the *Butyrivibrio* genus and the VFAs. Significant fold increases in *Streptococcus bovis* were shown during adaptation to the high-concentrate (high-grain) diet, whereas *Butyrivibrio fibrisolvens* and the populations gradually decreased as the animals adapted to the high-concentrate diet [67]. Additionally, *Butyrivibrio fibrisolvens* is known as another major bacteriocin producer in the rumen. Generally, in this study, the changes in the populations of studied microorganisms did not correlate with the changes in the rumen fermentation products. This was probably caused by the fact that the OEs used at the tested concentrations promoted the growth not only of *Streptococcus bovis* but also of bacteria that were dominant in the rumen (e.g., *Prevotella* spp.). The growth of other bacteria stimulated by *Cannabis* EOs supplementation was most likely responsible for the observed increase in total VFAs. The noted alterations will be the subject of our future studies. No changes in lactate production and no decrease in pH may suggest that the bacterial composition has high buffering abilities. Other bacteria, such as *Megasphaera elsdenii* and *Prevotella bryantii*, were involved in the utilisation of lactate and, at the same time, in the production of VFAs. However, our research indicates that it is possible to reduce the concentration of bacteria responsible for ruminal acidosis with the use of the EOs of *Cannabis* species through a reduction in *Lactobacillus* spp. However, in the case of *S. bovis* bacteria, much higher concentrations of EOs would probably be required to induce the inhibition of their growth.

## 4. Materials and Methods

### 4.1. Chromatographical Analysis

Essential oils (EOs) were obtained by steam distillation of the inflorescences of the *Cannabis sativa* Finola variety, harvested in 2022, and *C. indica* using a Deryng apparatus in Lab4Tox, Wrocław, Poland (Laboratory for Toxicological Forensic Studies). Analysis of the essential oils was performed using a Shimadzu 2020 GC-MS single-quadrupole chromatograph equipped with an SH-5 MSi capillary column (30 m × 0.25 mm × 0.25 µm; Shimadzu, Kyoto, Japan). The dried anhydrous sodium sulphate (VI) essential oil (10 mL) was dissolved in dichloromethane (CRCH sp. z o.o., Wrocław, Poland). The injection volume was 1 µL (220 °C, split ratio 50, with helium as the carrier gas at a flow rate of 1 mL·s^−1^).

The GC oven temperature programme started at 50 °C and was held for 2 min; then, it was increased to 160 °C at a rate of 1 °C·min^−1^; afterwards, it was increased to 300 °C at a rate of 15 °C·min^−1^ and held for 10 min. The total time of the programme was 129 min and 33 s. The MS operational conditions were as follows: ion source temperature: 250 °C, interference temperature: 250 °C, and scanned *m*/*z* range: 35–400. Supplementary Materials S3: GC-MS data. 

The chiral analysis was performed using the same chromatograph, equipped with a Cyclodex column (50 m × 0.25 mm × 0.25 mm), Agilent J&W, Santa Clara, CA, USA. The GC oven temperature programme started at 45 °C; then, it was increased to 200 °C at a rate of 3 °C·min^−1^; finally, it was raised to 220 °C at a rate of 25 °C·min^−1^ and held for 1 min. The total time of the programme was 53 min and 47 s. 

The following methods were used to identify the components of the EOs: (1) a comparison of the obtained mass spectra with the databases NIST 23 (National Institute of Standards and Technology) and FFNSC (the mass spectra of flavours and fragrances of natural and synthetic compounds); (2) a comparison of the calculated linear retention indices (LRIs) using a retention indices calculator with values presented in NIST 23 and FFNSC; and (3) a comparison of the retention times of the unknown compounds with authentic standards. Two software packages were used for the analysis: AMDIS (v. 2.73) and the ACD/Spectrus Processor v. 2021.2.1 (Advanced Chemistry Development, Toronto, ON, Canada). Enantiomeric excess was expressed in percentages.

The volatile fatty acids were analysed using gas chromatography coupled with MS spectrometry (Saturn WorkStation 2000/2000) and a WAX polar column (30 m × 0.25 mm × 0.25 μm, Phenomenex, US, with the parameters of the GC column). The temperature ramp was as follows: 50 °C was held for 4 min, then the temperature was increased by 10 °C min^−1^ to achieve 180 °C. The split ratio was set at 20, and the injector temperature was 220 °C. The quantification was based on the calibration curves of the VFA standards.

The methane level was analysed after incubation using a gas chromatography method. The gas was collected for the CH_4_ analysis using the Hamilton 1700 series with a 1 mL gas-tight syringe from a closed bottle through a side port after the fermentation was complete. During the sampling, the syringe was flushed with the produced gas to ensure that a homogeneous sample was collected. The samples were injected directly into the column for analysis using a gas chromatography method (Shimadzu GC-2030 equipped with a thermal conductivity detector (TDC)), a flame ionisation detector (FID), and a non-polar PLOT column, the Shimadzu SH-Q-BOND, DF 10, with a temperature range from −60 to 280/300 °C (Kyoto, Japan), with two replicates per treatment.

### 4.2. Ruminal Inoculum and In Vitro Incubation

Ruminal fluid was collected through cannulas from non-lactating Polish Holstein-Friesian cows of the black and white variety, weighing approximately 650 ± 25 kg in BW. The dry matter intake of the feed ratio was 11 kg·d^−1^. The composition of the feed ration was as follows: grass silage (70% DM), pasture hay (16% DM), barley ground (7.5% DM), rapeseed meal (5.5% DM), and premix (0.986% DM). Fresh water was provided ad libitum.

The rumen contents were collected through the fistula of the rumen-cannulated cows in the amount of 3 L before morning feeding and were placed in thermoses with an internal temperature of 39 °C. Before use, the rumen fluid was filtered through a three-layer cheesecloth. The experimental procedures were conducted in accordance with European Union Directive 2010/63/EU, made on 22 September 2010 [68]. The cow cannulation was approved by the Local Ethics Committee on Animal Experiments (Protocol No. 053/2019).

The fermentation processes were carried out using the Ankom RF gas production system (ANKOM Technology, Macedon, NY, USA), which consisted of glass bottles equipped with temperature (5–60 °C) and pressure (from −69 to +3447 kPa) sensors (resolution: 0.27 kPa; accuracy 0.1%), under anaerobic conditions at a temperature of 39 °C [65]. Six biological samples were prepared for the incubation experiments. Each biological sample was homogenised and mixed with pre-warmed solution in a 39 °C buffer [69] in a 1:4 ratio (50 mL of rumen fluid and 200 mL of buffer) and transferred to pre-warmed glass bottles with a total volume of 250 mL. The biological samples were divided into the following groups: control (Con), with the addition of 1 mg of monensin (Mon), the addition of 50 µL of EO distilled from *C. sativa* (marked as *C. sativa* 50), the addition of 100 µL of EO from *C. sativa* (*C. sativa* 100), the addition of 50 µL of EO from *C. indica* (*C. indica* 50), and the addition of 100 µL of EOs from *C. indica* (*C. indica* 100). To each sample, 1 g of a mixture of wheat and grass feed (1:1) was also added. Samples for VFA, lactate, and microbiological analysis were taken before the incubation and after 6 and 24 h.

The bottles were tightly closed and washed with CO_2_ through a Luer port to achieve anaerobic conditions until the internal pressure exceeded 8 psi. Once the gases were released from the bottle, an automatic real-time recording of gas production was initiated. During fermentation, the bottles were kept in a shaking water bath at 39 °C to ensure that the solution was at a stable temperature during the measurements. After 24 h of incubation, the cumulative gas pressures were read from the Ankom RF instrument dataset.

The culture was subsampled for the analysis of pH (Mettler-Toledo, MP230, Columbus, OH, USA), lactate concentration, volatile fatty acid (VFA) concentration, and DNA extraction. In the lactate analysis, the centrifuged liquid was diluted (1:40) in distilled water. Lactate concentrations were determined using an ABX reagent for the Horiba Pentra 400 analyser (Horiba-ABX Diagnostics, Grabels, France) [70]. The methane level was measured after incubation using a gas chromatography method. The following formula was used to calculate the CH_4_ yield (in mL) [71]:

$${\mathrm{CH}}_{4}=\frac{{\mathrm{percentage}\;\mathrm{concentration}\;\mathrm{of}\;\mathrm{CH}}_{4}\cdot \mathrm{gas}\;\mathrm{produced}\;\mathrm{in}\;\mathrm{mL}}{100}$$

The liquid contents of the samples were analysed to determine the overall concentration of VFAs and the percentage of individual fatty acids using a gas chromatograph (Varian Saturn WorkStation 2000/2000; USA). Based on an analysis of the level and profile of the VFAs; the VFA utilisation index (NGR) of the volatile fatty acids; expressed as the ratio of non-glucogenic to glucogenic VFAs; was calculated according to the following formula [72]:

$$\mathrm{NGR}=\frac{\left(A+2B+\mathrm{Bc}\right)}{\left(P+\mathrm{Bc}\right)\;}$$

The fermentation efficiency coefficient was calculated according to the formula given below [73]:

$$\mathrm{FE}=\frac{\left(0.622A+1.092P+1.56B\right)\cdot 100}{\left(A+P+2B\right)}$$

where A, P, and B are the percentage contents in the VFA total concentration of the tested acetic acid, propionic acid, and butyric acid, respectively, while Bc is the percentage of the total concentration of VFAs (valeric acid and branched short-chain fatty acids).

### 4.3. DNA Extraction and Analysis of Changes in the Composition of Microorganisms Using qPCR

The total genomic DNA isolation of the bacterial strains obtained from the rumen fluid was performed using the Masterpure Complete DNA and RNA Kit (Lucigen, Madison, WI, USA) according to the manufacturer’s instructions. The concentration and purity of the extracted DNA were measured using a spectrophotometer (DS-11 FX, DeNovix, Wilmington, NC, USA) at 280, 260, and 230 nm. The bacterial strains obtained from the rumen fluid were subjected to total genomic DNA isolation using the Masterpure Complete DNA and RNA Kit (Lucigen, Madison, WI, USA) as per the manufacturer’s instructions. The extracted DNA was measured for concentration and purity using a spectrophotometer (DS-11 FX, DeNovix, Wilmington, NC, USA) at 280, 260, and 230 nm. The extracted DNA was standardised to 50 ng·μL^−1^ for qPCR.

Microbiological analyses were performed according to the method employed by Abdelmegeid et al. [74]. The standards were obtained using dilutions of purified genomic DNA extracted from pure cultures (PCM 2092 *Streptococcus bovis*, PCM 2379 *Lactococcus lactis*, and CCUG 35459T *Butyrivibrio fibrisolvens*) with a known concentration. The semiquantitative relative change in the abundance of target microorganisms was estimated using qPCR amplification of the 16S rDNA genes. The qPCR was carried out on the CFX Connect™ Real-Time System (Bio-Rad, Herkules, CA, USA), using SsoFast EvaGreen Supermix (Bio-Rad, Herkules, CA, USA). The reaction mixture contained 1 µL of DNA, 0.5 μM of each primer (see Table 3), 10 µL of SsoFast EvaGreen Supermix, and water (up to 20 µL). The reaction mixtures were initially incubated for 30 s at 95 °C, followed by 40 cycles at 95 °C for 10 s and at 58 °C for 15 s. The specificity of the PCR was evaluated using melt curve analysis in the temperature range from 95 to 58 °C for each reaction. The negative controls without template DNA, standards, and samples were analysed in triplicate. The relative abundance of the bacterial species was calculated using the geometric mean of two universal primers with the efficiency-corrected Δ−CT method. Thus, the abundance of each target bacteria was determined relative to the overall abundance of the total bacteria measured with the universal primers. The change in quantity was presented as a percentage of the change in a target bacterium after 24 h in comparison to time 0.

### 4.4. Statistical Analysis

The results were subjected to statistical analysis using a one-way analysis of variance (ANOVA) in the Statistica 13.3 program. The analysis was preceded by the evaluation of all the data for normality using the ANOVA procedure. The significance of the differences between the experimental groups was determined using Tukey’s test for the analysis of VFAs. The analysis data for each group were represented by three analytical repeats for each biological sample. The statistical model was as follows:

Yijk=μ+α+β+ε

where Yijk is the experimental data, μ is the overall mean, α is the random effect of the fermentation trials (i = 1 to 2), β is the fixed effect of the dietary treatments (j = 1 to 6), and ε is the residual error.

The significance in the relative bacterial abundance differences in response to the treatments was evaluated using ANOVA and the Bonferroni post hoc test. The results were considered statistically significant at *p* < 0.05.

## 5. Conclusions

The in vitro studies indicated that the supplementation of essential oils extracted from *Cannabis* spp. can significantly affect the population of selected rumen bacteria and modify the production of VFAs. The proposed addition of hemp EOs limited the growth of *Lactobacillus* spp., and higher concentrations exhibited no negative effects on *Butyrivibrio* spp. No inhibition of *S. bovis* growth was observed. The addition of EOs from *C. sativa* caused an increase in total VFAs, as well as acetate, at the end of the incubation of rumen fluid. However, *C. indica* at a higher concentration resulted in a significant increase (*p* < 0.01) in acetate and propionate, but this was only after 6 h of incubation. After this time, the propionate and acetate decreased; however, these changes were temporary. Therefore, they depended on the supply of active compounds and the rate of metabolisation over time by these bacteria and other microorganisms not tested in this experiment. The applied EOs had a greater impact on the reduction in methane production after 6 h, but no apparent effect after 24 h was observed.

Additional studies should be conducted to investigate the feasibility of using *Cannabis sativa* or *Cannabis indica* as a feed additive and to determine the optimal level or form of supplementation in ruminants.

## Figures and Tables

**Table 1:** Chemical composition of essential oils distilled from *Cannabis sativa* and *Cannabis indica*.

No.	Peak Name	KI Exp.	KI Lit.	CAS	Identification	*C. sativa*	*C. indica*
1	2-octene, (E)-	810	815	13389-42-9	MS, KI	0.043	0.045
2	4-Hydroxy-4-methyl-2-pentanone	843	842	123-42-2	MS, KI	0.266	0.194
3	Tricyclene	919	923	508-32-7	S, MS, KI	0.395	0.212
4	α-Thujene	928	927	2-05-2867	S, MS, KI	0.192	0.114
5	α-Pinene	935	933	80-56-8	S, MS, KI	**7.243**	**8.892**
6	Camphene	950	953	79-92-5	S, MS, KI	0.272	0.172
7	Sabinene	974	972	3387-41-5	S, MS, KI	0.05	0.036
8	β-Pinene	976	978	127-91-3	S, MS, KI	4.148	4.081
9	Myrcene	992	991	123-35-3	S, MS, KI	**13.772**	0.084
10	Ethyl hexanoate	1001	1003	123-66-0	S, MS, KI	0.051	0.362
11	*p*-Mentha-1(7),8-diene	1009	1004	499-97-8	MS, KI	0.221	1.362
12	Hexyl acetate	1017	1012	142-92-7	S, MS, KI	0.085	0.296
13	*p*-Cymene	1024	1025	99-87-6	S, MS, KI	0.542	0.265
14	Limonene	1029	1030	138-86-3	S, MS, KI	7.273	2.22
15	Eucalyptol	1030	1032	470-82-6	S, MS, KI	0.892	0.147
16	β-Ocimene (Z)-	1041	1035	3338-55-4	S, MS, KI	0.145	1.064
17	β-Ocimene (E)-	1051	1046	3779-61-1	S, MS, KI	0.655	9.778
18	Melon aldehyde	1055	1053	106-72-9	MS, KI	0.088	0.004
19	γ-Terpinene	1059	1058	99-85-4	S, MS, KI	0.031	0.238
20	*cis*-Sabinene hydrate	1067	1069	15537-55-0	S, MS, KI	0.108	0.097
21	Fenchone	1085	1090	1195-79-5	S, MS, KI	0.037	0.001
22	Terpinolene	1086	1086	586-62-9	S, MS, KI	0.449	**12.267**
23	6,7-Epoxymyrcene	1093	1096	29414-55-9	MS, KI	0.082	0.031
24	Linalool	1099	1101	78-70-6	S, MS, KI	2.064	0.176
25	Heptenyl acetate	1109	1104	6939-73-4	MS, KI	0.616	0.089
26	*trans*-Pinene hydrate	1117	1121	4948-29-2	MS, KI	0.383	0.059
27	*trans*-Sabinol	1135	1140	471-16-9	MS, KI	0.056	0.05
28	Myroxide (*E*)	1145	1141	28977-57-3	MS, KI	0.076	0.267
29	β-Pinene oxide	1159	1156	6931-54-0	MS, KI	0.041	0.05
30	Isoborneol	1162	1165	10385-78-1	S, MS, KI	0.111	0.018
31	Linalool ethyl ether	1174	1166	72845-33-1	MS, KI	0.366	0.198
32	*p*-Cymen-8-ol	1183	1189	1197-01-9	S, MS, KI	0.416	0.587
33	Santolinyl acetate	1187	1175	79507-88-3	MS, KI	0.429	0.098
34	Hexyl butyrate	1194	1195	2639-63-6	MS, KI	0.363	0.121
35	Ethyl octanoate	1199	1202	106-32-1	MS, KI	0.082	0.026
36	*cis*-4 Caranone	1209	1205	06.01.4176	MS, KI	0.041	0.076
37	*trans*-Carveol	1216	1223	1197-07-5	MS, KI	0.066	0.066
38	Carvone	1240	1246	99-49-0	S, MS, KI	0.044	0.009
39	Dec-(5*Z*)-en-1-ol	1274	1263	51652-47-2	MS, KI	0.034	0.062
40	Phellandral	1278	1277	21391-98-0	S, MS, KI	0.055	0.103
41	*iso*-Dihydrocarveol acetate	1332	1328	160868-58-6	MS, KI	0.06	0.03
42	α-Cubebene	1345	1349	31141-66-9	S, MS, KI	0.04	0.007
43	α-Ylangene	1364	1371	14912-44-8	MS, KI	0.123	0.05
44	α-Copaene	1368	1375	138874-68-7	S, MS, KI	0.069	0.026
45	Longicyclene	1382	1372	1137-12-8	MS, KI	0.303	0.04
46	7-*epi*-Sesquithujene	1386	1387	159407-35-9	MS, KI	0.053	0.037
47	Hexyl hexanoate	1388	1390	6378-65-0	MS, KI	0.125	0.277
48	β-Elemene	1396	1390	33880-83-0	MS, KI	0.558	0.389
49	Sesquithujene	1402	1402	58319-06-5	MS, KI	0.115	0.05
51	*E*-β-Caryophyllene	1408	1413	87-44-5	S, MS, KI	**18.451**	**24.096**
52	α-Santalene	1413	1418	512-61-8	MS, KI	0.284	0.3
53	γ-Elemene	1427	1432	29873-99-2	MS, KI	0.885	0.265
54	α-*trans*-Bergamotene	1431	1432	13474-59-4	S, MS, KI	**3.126**	**2.354**
55	α-Humulene	1443	1445	24568-69-2	S, MS, KI	**6.191**	**7.702**
56	Aromadendrene	1447	1440	109119-91-7	MS, KI	0.375	0.842
57	Sesquisabinene	1457	1455	58319-04-3	MS, KI	**1.973**	**2.648**
58	Valerena-4,7(11)-diene	1467	1455	351222-66-7	MS, KI	0.141	0.16
59	Dauca-5,8-diene	1469	1473	142928-08-3	MS, KI	0.133	0.019
60	9-epi-(*E*)-Caryophyllene	1474	1464	68832-35-9	MS, KI	1.791	1.946
61	α-Curcumene	1478	1480	644-30-4	MS, KI	0.412	0.19
62	β-Selinene	1483	1492	17066-67-0	MS, KI	1.298	1.34
63	unknown ^a^	1486	–	–	–	0.004	0
64	α-Bulnesene	1496	1505	489-81-6	MS, KI	1.029	0.16
65	β-Bisabolene	1503	1508	4891-79-6	MS, KI	0.776	0.389
66	γ-Patchoulene	1507	1506	508-55-4	MS, KI	1.241	0.896
67	δ-Cadinene	1515	1518	16729-01-4	S, MS, KI	0.093	0.074
68	β-Sesquiphellandrene	1518	1523	20307-83-9	MS, KI	0.13	0.221
69	γ-Cuprenene	1523	1530	4895-23-2	MS, KI	3.078	0.895
70	7-epi-a-Selinene	1529	1518	123123-37-5	MS, KI	**4.188**	1.591
71	α-Bisabolene (*E*)-	1540	1540	25532-79-0	MS, KI	1.128	0.268
72	Selina-3,7(11)-diene	1544	1546	222187-60-2	MS, KI	2.09	0.566
73	(*E*)-Nerolidol	1562	1561	40716-66-3	S, MS, KI	0.138	0.26
74	Caryophylene oxide	1569	1576	1209-61-6	MS, KI	**4.198**	**4.674**
75	Spathulenol	1575	1578	72203-24-8	MS, KI	0.09	0.015
76	Viridiflorol	1588	1594	19078-39-8	S, MS, KI	0.19	0.088
77	Caryophyllene oxide	1594	1587	1139-30-6	S, MS, KI	1.004	1.125
78	Fokienol	1597	1596	33440-00-5	MS, KI	0.166	0.012
79	Guaiol	1603	1603	13822-35-0	MS, KI	0.196	0.075
80	Humulene epoxide II	1613	1613	19888-34-7	MS, KI	0.143	0.084
81	*allo*-Aromandendrene epoxide	1646	1644	85760-81-2	MS, KI	0.208	0.073
82	Agarospirol	1656	1646	1460-73-7	MS, KI	0.086	0.002
83	*Allo*-himachalol	1660	1664	19435-77-9	MS, KI	0.101	0.104
84	*epi*-α-Bisabolol	1676	1679	23178-88-3	MS, KI	0.193	0.017
85	Mayurone	1706	1711	4677-90-1	MS, KI	0.207	0.027
86	1,10-Dihydronootkatone	1753	1751	20489-53-6	MS, KI	0.063	0.017
87	*m*-Camphorene	1956	1946	20016-73-3	MS, KI	0.291	0.888
88	*p*-Camphorene	1987	1984	20016-72-2	MS, KI	0.135	0.496
89	Geranyllinalool	2031	2034	1113-21-9	MS, KI	0.07	0.01
90	Cannabidiol	2438	2441	13956-29-1	MS, KI, S	tr	0.03

Abbreviations: MS—mass spectrum; S—standard of compound; KI—Kovats retention index according to NIST23 database; ^a^—MS spectrum of compound is presented in Supplementary Materials Figure S1; GC-MS chromatograms of (a) *C. sativa* and (b) *C. indica* are presented in Supplementary Materials Figure S2; tr—trace < 0.01.

**Table 2:** Effects of essential oils of *Cannabis sativa* and *Cannabis indica* on in vitro fermentation characteristics.

Parameters	Treatments ^a^						SEM	*p*-Value
	Con	Mon	*C. sativa* 50	*C. indica* 50	*C. sativa* 100	*C. indica* 100		
pH								
6 h	6.69	6.69	6.72	6.71	6.70	6.77	0. 011	0.235
24 h	6.61	6.63	6.63	6.63	6.62	6.67	0.007	0.319
Total VFAs								
6 h	75.24A	75.38A	79.94B	70.60C	70.87C	83.88C	0.021	0.001
24 h	83.39A	85.77A	93.20B	88.08B	93.99A	67.12B	0.106	0.001
Acetate								
6 h	52.11 ^A^	50.81 ^A^	58.30 ^B^	54.03 ^B^	51.55 ^AB^	60.12 ^B^	0.085	0.001
24 h	59.48 ^A^	58.66 ^A^	69.06 ^B^	62.14 ^A^	67.89 ^B^	48.08 ^A^	0.169	0.001
Propionate								
6 h	20.83 ^A^	22.26 ^A^	19.34 ^AC^	14.27 ^B^	17.02 ^BC^	21.46 ^A^	0.073	0.001
24 h	14.66	16.78	14.10	15.01	14.97	14.62	0.033	0.283
Butyrate								
6 h	17.97 ^A^	15.07 ^BC^	13.71 ^BC^	13.46 ^BC^	16.17 ^C^	15.24 ^BC^	0.039	0.001
24 h	27.90	23.68	28.62	26.32	28.57	28.90	0.033	0.310
Acetate to propionate ratio								
6 h	2.54 ^AC^	2.28 ^A^	3.01 ^B^	3.79 ^B^	3.03 ^B^	2.80 ^BC^	0.118	0.001
24 h	4.11 ^A^	3.54 ^A^	4.90 ^B^	4.14 ^A^	4.54 ^B^	3.29 ^A^	0.147	0.001
Fermentation efficiency, %								
6 h	76.45 ^A^	76.86 ^A^	74.95 ^B^	73.65 ^BC^	75.14 ^B^	75.43 ^B^	0.264	0.001
24 h	80.63	79.39	81.00	80.33	80.39	80.43	0.246	0.189
NGR								
6 h	4.59 ^A^	4.97 ^A^	5.72 ^B^	5.50 ^A^	4.82 ^A^	5.49 ^B^	0.095	0.001
24 h	5.63	5.16	6.38	5.72	6.08	4.93	0.223	0.041
Methane, mL·L^–1^								
6 h	32.41 ^A^	24.96 ^B^	28.25 ^A^	29.28 ^A^	39.50 ^A^	23.44 ^B^	2.362	0.052
24 h	40.70 ^A^	29.80 ^B^	58.79 ^BC^	46.90 ^A^	55.43 ^B^	56.30 ^B^	2.624	0.001
Lactate, mmol·L^–1^								
24 h	7.62	6.18	6.72	6.75	6.89	6.81	0.382	0.127

^A, B, C^—the means in a row followed by different letters are significantly different (*p* < 0.01).; SEM—the standard error of the mean, VFA—volatile fatty acid. ^a^ Treatments were as follows: Con—control; Mon—1 mg of monensin; *C. sativa* 50—50 µL of EO from *C. sativa; C. sativa* 100—100 µL of EO from *C. sativa*; *C. indica* 50—50 µL of EO from *C. indica*; *C. indica* 100—100 µL of EO from *C. indica*; NGR—ratio of non-glucogenic to glucogenic VFAs.

**Table 3:** Species-specific bacterial qPCR primers.

Bacterial Strain	Primer Sequence for (5′-3′)	Primer Sequence Revers (5′-3′)	Reference
*Lactobacillus* spp.	CTCAAAACTAAACAAAGTTTC	CTTGTACACACCGCCCGTCA	[75]
*Streptococcus bovis*	TTCCTAGAGATAGGAAGTTTCTTCGG	ATGATGGCAACTAACAATAGGGGT	[76]
*Butyrivibrio fibrisolvens*	TAACATGAGTTTGATCCTGGCTC	CGTTACTCACCCGTCCGC	[77]
Bacteria general 1	GGATTAGATACCCTGGTAGT	CACGACACGAGCTGACG	[78]
Bacteria general 2	GTGSTGCAYGGYTGTCGTCA	ACGTCRTCCMCACCTTCCTC	[79]

## Data Availability

The data presented in this study are available upon request from the corresponding author.

## Supplementary Materials

The following supporting information can be downloaded at: https://www.mdpi.com/article/10.3390/ijms25115861/s1.
