# Supplementary material for: Comparison of the Effects of Essential Oils from Cannabis sativa and Cannabis indica on Selected Bacteria, Rumen Fermentation, and Methane Production—In Vitro Study
Source: Int J Mol Sci. 2024 May 28;25(11):5861. doi: 10.3390/ijms25115861 (PMC11172183; doi:10.3390/ijms25115861)
Supplement: Supplementary file 1 [file ijms-25-05861-s001.zip › ijms-2964595-supplementary.pdf]

Supplementary materials

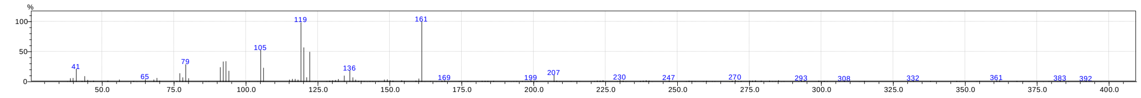

Figure S1. MS chromatogram of unknown sesquiterpene compound with RI 1486

(**a**)

(**b**)

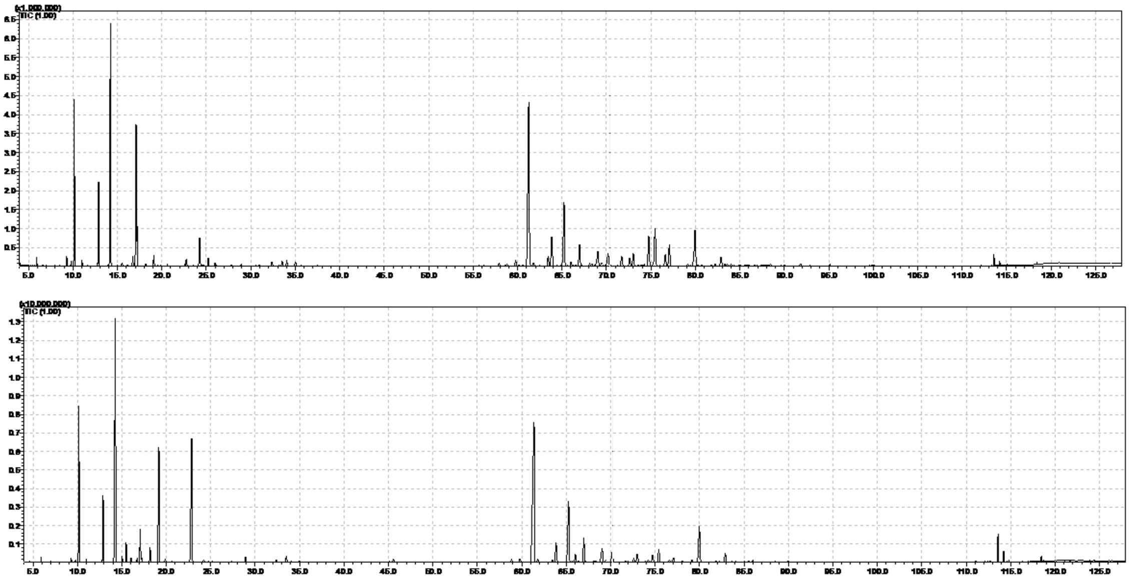

Figure S2. GC-MS chromatograms of **(a)** *C. sativa* and **(b)** *C. indica*

S3. Link to source GC-MS data

https://drive.google.com/file/d/17Exdfw2snScbVSvKZTnydUnG14JU9loc/view?usp=drive_link, https://drive.google.com/file/d/17Fc3E365FGs1Hg_tyiE39--dbqiW4QYF/view?usp=drive_link
